# Aspirations and the subjective future of migration: comparing views and desires of the “time ahead” through the narratives of immigrant domestic workers

**DOI:** 10.1186/s40878-016-0047-6

**Published:** 2017-02-10

**Authors:** Paolo Boccagni

**Affiliations:** University of Trento, via Verdi 26, Trento, 38122 Italy

**Keywords:** International migration, Aspirations, Future, Domestic work, Narratives, Italy

## Abstract

Migrants’ aspirations are a meaningful and under-appreciated research subject. My paper investigates their development and implications over the life course, building on an archive of life stories of immigrant domestic workers in Italy. It dissects the biographical bases of aspirations as ways of cultivating open representations of the future; hence, as a window on migrants’ potential to shape the future itself, given their assets, the external structure of opportunities and the relational fields in which they are embedded. Migrants’ views and desires about the future, as individuals and members of families and broader communities, evolve in parallel with their biographies. Over time, they face “reality checks” which may make them elusive, opening up to unintended social consequences. Immigrant domestic workers in Italy are a case in point. What these interviewees reportedly aspired *then*, while leaving home, may significantly differ from what they do aspire *now*; a gap which is telling of their often limited scope to negotiate a way across local and transnational life milieus. I reconceptualise this gap in aspirations, and in their accomplishment, in terms of “contents”, “references” and “horizons”. How and why migrant aspirations are transformed over time, and how different kinds of aspirations impinge on their life trajectories, are questions that generate fruitful insights for migration studies.

## Introduction

Migrants’ aspirations, as purposeful constructions of the future which evolve over time, are a fascinating and relatively neglected research subject. This paper aims to advance the debate on them, through a collection of biographical interviews which illuminate the interdependence between personal memories of the past, present life conditions and subjective constructions of the future. My focus is, retrospectively, on the ways in which future-related aspirations are cultivated and tentatively enacted over a biographical continuum in which international migration marks a major discontinuity. I build on an archive of life stories of 224 immigrant domestic workers in Italy, part of a broader study conducted all over the country between 2005 and 2008 (Catanzaro & Colombo, 2009).

In my understanding, aspirations are emotionally thick representations of what one’s future *might* and *should* look like, given the present circumstances and the experience of the past as re-codified from the “here-and-now”. Judging from the literature available, migrants’ imagined constructions of their future – as individuals and members of families and broader communities – are quite understudied. This is unfortunate, since revisiting their biographical narratives in this optic can illuminate the subjective and emotional drivers of their life trajectories overseas, under a twofold pressure: the multi-sited relational settings in which their life projects are embedded, and the changing external structures of opportunities.

Through a content analysis of immigrant domestic workers’ narratives, with a focus on their mixed stances towards the future, I examine the interface between their reported aspirations and their evolving life and work circumstances. How (far) do the views and constructions of the future shape the biographical trajectories of these individuals? And how are such views and constructions affected by migrants’ life conditions over time?

In order to address these questions I first review the relevant literature, at the intersection between the study of aspirations and of the social meanings of the future. After a brief presentation of my case study, I advance a heuristic frame for charting migrants’ reported aspirations in light of the interdependence between the influences of the past, the structure of opportunities of the present and the construction of the future as a more or less open social field. Respondents’ self-narratives are analysed as a source of insight on their evolving ways of cultivating, and to some extent achieving, different kinds of aspirations.

As it turns out, their future-related views and imaginations face reality checks which may make them elusive and open up to unintended social consequences, including the systematic postponement, “downsizing” or “displacement” of the initial migration aims. This leads me to revisit the very concept of aspirations, as a conflation of different *contents*, *relational references* and *space-time horizons*. Whether migrants’ imagined and desired futures turn into reality or not, a retrospective focus on them is critical to make sense of their evolving attitudes and life projects vis-à-vis both host and home societies.

## Aspirations as goal-oriented cognitions and subjective glimpses into the future

### A faceted and interdisciplinary research field

Approaching migrants’ reported aspirations over their life trajectories requires, first of all, an overview of the main uses of this notion in social research. Its meaning and implications are less obvious than its discursive currency would suggest.

The concept of aspiration has gone through an extended, if discontinuous intellectual career over the last decades. Much of its use as an empirical construct has involved research on social mobility, education and employment. A number of longitudinal studies have been done, particularly in the US, on the attitudes and motivations underlying the younger generations’ educational and occupational pathways (Haller, Otto, Meier, & Ohlendorf, 1974). The social production of the youth’s subjective motivations and expectations, as a predictor – combined with external circumstances – of their occupational trajectories, has been investigated through qualitative fieldwork too. A celebrated instance is MacLeod’s (2009) ethnography of the schooling and employment trajectories of two marginal youth groups, in light of their distinctive constellations of future life-goals, as they were emerging and reproduced in their everyday lives. Earlier elaboration on the concept of aspiration can however be found in Lewin’s field theory, as a starting point of a tradition in social psychology that has referred to aspiration as “the cognitive orientational aspect of goal-directed behaviour” – or more simply, as “ego’s own orientation to a goal” (Haller, 1968, p. 484). This cognitive and orientational basis is not to deny, of course, the central influence of “ability and circumstances” (Haller, 1968, p. 486) on individuals’ attainment, as a more or less imperfect accomplishment of their initial aspirations.

Sociologically speaking, aspirations are a valuable research field on the interaction between structure and agency; put differently, between mutually interconnected structural factors (i.e. family backgrounds, education, social class, employment etc.) and individual orientation to social action. It is however on the side of individual preferences and inclinations that a focus on aspirations – as distinct from expectations (Spenner & Featherman, 1978) – sheds light. Aspirations could be defined as the personal “ability to believe in «making it»” in a given life environment (Chamberlain, 2001, p. 8), in light of the subjective perception of it and of one’s evolving biographical circumstances. Such a background is particularly complex when it comes to migrants’ aspirational trajectories, as it may involve different locations and relational settings over time and space.

The concept of aspirations is relatively novel, instead, as a specific topic of analysis for migration studies. The most oft-quoted contribution in this respect, Portes, McLeod, and Parker (1978) “Immigrant aspirations”, provides a survey-based map of newcomers’ aspirations in three respects – employment, income, education. More recently, in the European context, explicit reflections include Carling’s (2002, 2014) argument on “migration aspirations”, basically framed as individuals’ “preference for migration over staying, regardless of the reason”; and De Haas (2011) theorization of aspirations as an individual requirement, along with “capabilities” (and “within a given set of opportunity structures”), for migration to start and be perpetuated over time. In terms of empirical research, some attempts have been made to investigate the determinants of migrants’ aspirations through quantitative techniques (e.g. Czaika & Vothknecht, 2012), and to classify them via qualitative ones (Bal & Willems, 2014; Van Meeteren, 2010; Van Meeteren, Engbersen, & Van San, 2009). Still another variation on the theme, out of migration studies and curiously disconnected from it, has been introduced by Appadurai’s (2004) “capacity to aspire” as a fundamental meta-objective of anti-poverty and development policies.

Still missing, though, is a systematic connection between the study of migration aspirations and temporalities – what this paper specifically aims at, starting from the subjective constructions of future that emerge out of migrants’ aspirations.

### The future as a shifting horizon for migrants’ aspirations

Across this wide and interdisciplinary spectrum of uses, relatively little attention has been paid to the temporal dimension of aspirations, in a dual sense: related to their evolution over time (as narratively reconstructed from the present), and to their interdependence with future-related views and desires at an individual and group level.^1^ This is unfortunate, since the conceptual promise of aspiration lies exactly in being a synthesis of the future-orientation of individual agency, as it evolves over time – in this case, parallel with migrants’ life trajectories. This by no means denies the challenges and dilemmas inherent in cultivating, and possibly realizing, one’s aspirations.

A time-sensitive understanding of aspirations, I contend, is particularly fruitful in migration studies. At first glance the connection between aspirations and migration is straightforward, even tautological. At its outset, labour migration can be appreciated as a tentative enactment of distinctive “capacities to aspire”; put differently, an intransitive effort at obtaining something more, whatever the ways of defining it and the definitional clarity. The perceived potential to achieve better life conditions is a motivational driver that reflects the distinctive aspirations of prospective leavers. Nevertheless, how the interaction between aspirations and extended mobility is initially framed, and how it evolves over time, is a matter for empirical exploration – just as I do in this paper.

Surprisingly, the future – as a field open to social imagination and (to a variable extent) initiative from the present (Adam & Groves, 2007; Luhmann, 1976) – is quite marginal from the debate on aspirations and, overall, from migration studies (among the exceptions, Cwerner, 2001; Griffiths, Rogers, Anderson, 2013). A case can be made, though, for labour emigration to stem also from an interiorized and socially legitimated view of the future as amenable to progress – of course, on a specific condition: that the bet is made to go and live elsewhere for an expectedly limited, but typically undeterminable time span. Where, with whom and in which life realms these future-oriented views do accomplish themselves is, again, an empirical question.

Following this premise, aspirations are not assumed here as an “object” to be measured and compared between different individuals or reference groups; for instance, migrants vs non-migrants (Czaika & Vothknecht, 2012). Whether an aspiration differential between movers and stayers exists, even prior to migration, is a question out of the purview of this article. Moreover, a comparison along similar lines should address what seems to be a widespread research finding: the modest “net causal efficacy” of ambitions and aspirations on individual life achievements over time, compared with major structural, relational and situational factors, including the main “contingencies” in the life course (Spenner & Featherman, 1978, p. 408).

More promisingly, individually displayed and reported aspirations are interrogated as a window into migrants’ constructions of their future, as affected by past life circumstances and by their ex-post redefinitions in terms of memories, recollections and rituals. Aspirations are approached as an expression of the self to which a certain view of the future – as elicitor of personal desires – is attached; and which results in distinctive (not necessarily consistent or consequential) social practices. Underlying this perspective is an understanding of migrants’ subjective future as a relatively manipulable field; hence, an object of projects with a potential to be fulfilled, in presence of favourable conditions (Adam & Groves, 2007).

Building on a number of narrative accounts of immigrant domestic workers in Italy, now, I explore how these future-related stances change over time, affecting and being affected by the changing life backgrounds associated with migration. Respondents’ aspirations emerge from the standing point of a “present” generally marked by limited and subordinate inclusion in the receiving country. Even so, the life stories of these interviewees point to a variety of aspirational contents and references, to be empirically classified and then theoretically revisited in this article.

## Reconstructing aspirations through biographical narratives: a case study of immigrant domestic workers in Italy

Empirically speaking, this article builds on an archive of in-depth interviews with low-skilled and middle-aged immigrant domestic workers in Italy, collected all over the country between 2005 and 2008 (Catanzaro & Colombo, 2009). This qualitative survey has resulted in an unprecedented archive of narrative data on the life trajectories of immigrants employed in domestic work. This labour market sector, which accounts for 20% of foreign employment in the country (IDOS, 2015), has been relatively resilient to the after-2008 crisis (Saraceno, Sciortino, & Barban, 2013) and has been fueled by an increasing demand for personal care to the elderly, primarily in domestic environments (Ambrosini, 2013; Da Roit, 2007; Rusmini & Pasquinelli, 2015).

Several of the topics addressed during these interviews had to do with orientations to the future – in fact, aspirations – at different stages of the life course. The interview guide included open-ended questions on respondents’ views, projects and expectations about the future as seen at that moment, but also, retrospectively, as seen while leaving home. This makes for an open-ended and flexible framework in which interviewees’ aspirations often emerge tacitly and indirectly. The word itself may be marginal from their everyday language. Nonetheless, their self-narratives are replete with instances of aspirations being cultivated, experienced and, to some extent, accomplished.

Out of this 682-item archive I have selected for content analysis 224 interviews to adult first-generation newcomers, based on a length of stay of ten years at least, against an average of seven for all interviewees. I assume this time-span to be extended enough to cast light on the evolution of their aspirations, parallel to their local integration processes. A stay in Italy of ten years or more is sufficient to emphasize the gap between the expected temporariness of most of these migration projects and the migrants’ factual settlement. This selection criterion has resulted in a subset of respondents with significant concentrations along lines of gender (women being 86%), work regimes (65% non-resident, hourly paid domestic workers) and national backgrounds (the most recurring being Filipinos [about 17%], Poles [8%], Albanians [8%], Ukrainians [7%] and Peruvians [6%]). Their mean age was around forty. Most of them, particularly women, cultivated extended transnational family ties with significant others left behind, primarily children and partners. This also affects their aspirational structures – the “will to have a future together as a family” being crucially emphasized in the narrative of Filipina Mel, and of many other interviewees.

Methodologically speaking, these interviews were based on a long and detailed guide, but were conducted in ways which enabled respondents to tell spontaneously their own life stories, as long as they had some fit with it.^2^ My ex-post analysis is based, first of all, on in-depth reading of all the relevant interviews, which I have re-coded in order to select all contents related to ‘future’, ‘aspirations’, and their synonyms. With the help of Atlas.ti. I have then revisited these excerpts, in light of the biographical and work circumstances of each interviewee. While I can produce only a few exemplary quotes in this paper, my overall argument builds on the much richer material which I have collected and selected in this way. Of course, such a retrospective and indirect account is not without limits – not only because it builds on a non-representative sample,^3^ related to a specific immigrant profile. Besides that, my analysis cannot do justice to the diversity in respondents’ demographic and socio-economic backgrounds, and to the socio-cultural variation in their ways of conceiving and articulating aspirations. Nor can it fully grasp the determinants of respondents’ selective memories of their past (and of course, of their aspirations-in-the-past). Nonetheless, I found this archive invaluable as a source of first-hand data on the ways in which migrants’ aspirations are articulated, reshaped and projected ahead, parallel with their life course.

The focus of my analysis, as a result, is less on the influence of respondents’ national, ethnic or family backgrounds, than on a different question: the evolution of their aspirations over time, as they interact with their processes of local integration and transnational engagement. The key issues are what respondents’ aspirations reportedly looked like at the start of the migration experience; how they have evolved over time; what their current configuration is like; how they fare vis-à-vis migrants’ actual “achievements” and employment conditions in a low-skilled and segregated niche such as domestic work in Italy.

## Tracking migrants’ aspirations over time: continuities, ruptures, reframes

It is now time to see how respondents’ evolving aspirations mark and reflect the steps of their life trajectories, and how they feed into their perceived future prospects. I first borrow insights from the literature on “socially expected durations” (Merton, 1984) and on the social construction and production of the future (Adam & Groves, 2007, 2011; Luhmann, 1976). I then enter into the domestic workers’ narratives, by matching up the temporal ends of their aspirational field: *then*, as they were leaving, and *now*, after at least one decade abroad as labour immigrants. Their “past futures” should be revisited in light of the present ones, and possibly as a source of insights on their “future futures” (Jedlowski, 2013; Luhmann, 1976). In all of these respects, the section paves the way for investigating respondents’ aspirations and mixed views of the future.

### “The future cannot begin”,^4^ but its constructions change over time

Respondents’ declared or implicit aspirations are assumed, here, as a gateway into their “engagement with the future” (Adam & Groves, 2007), which migration itself epitomizes. Although such aspirations can be approached only as retrospective discursive representations, they are still telling of their attempts at, and limitations in, exerting control on the future life course. By analysing respondents’ reconstructions of their past experiences and their views of the future, I aim to make sense of their changing ways of conceiving the future and tentatively “emplacing” it in distinct life projects over time. With some simplification, their reported views of the future before leaving can be compared with those which paralleled the course of migration and with those manifested “at present”, while being interviewed. What is, then, the relation between migrants’ evolving life experience and the development of their aspirations? What accounts for their capability to cultivate them over time and to project them ahead, in light of their socio-demographics and of their groups of reference?

**Fig. 1:**
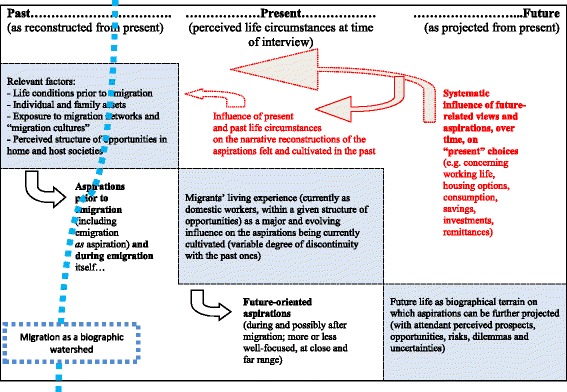
The development of migrant aspirations over time as a set of mutual influences: key factors emerging from migrants’ biographic narratives

A heuristic map of the key factors involved, and of their mutual, if asymmetrical influences is sketched out in Fig. 1. Assuming migration as a major watershed *[thick dotted line on the left]* in the life course of the interviewees, their life stories as reported in the present should be embedded in a variety of environmental and biographical factors, past and present *[grey squares]*. Such factors are the breeding ground for their future-oriented aspirations *[black arrows below]*. They are also shaped, however, by future-related influences *[clear-dotted arrows above]*, in a dual sense. Narratively speaking, the reconstruction of past life conditions and aspirations is affected by the circumstances of the present (i.e. the future-of-the-past). In turn, present life conditions and the attendant aspirations are affected by future-related dreams, fears, or concerns, which do impinge on migrants’ present life styles (see the examples under the “future” label in Fig. 1).

A complex set of interdependencies between past, present and future is, therefore, the background against which migrants’ aspirations should be appreciated. Doing so entails looking both at the ongoing influence of the external structure of opportunities, and at their changing potential and volition to achieve their aspirations. The latter, importantly, are time- and life course-dependent. At all of the stages depicted here, individuals’ aspirations are likely to vary at several levels: regarding the emplacement, the emotional bases and substantive contents, as well as the expected beneficiaries and individuals’ potential to achieve them. I return below on the need to unpack migrants’ aspirations along these lines, in light of my empirical material.

### The future seen from the past: tracing the roots of the aspiration to leave

Aspirations, in one form or another, underlie our everyday experience at any stage of the life course. Some biographic events, however, are particularly telling about the ways in which the future is seen, constructed and tentatively achieved. The start of emigration is arguably one of these events. Within my narrative analysis, it stands out as a focal point, first, for reconstructing migrants’ past futures as elicitors of aspirations; second, for comparing past aspirations with present achievements and, as critically, present, but future-oriented aspirations.

Investigating personal motivations for leaving is a reasonable way to approximate respondents’ aspirations – at least as (re)defined from the here-and-now. There is a striking commonality at the inception of the interviewees’ narratives, cutting across their different contexts of origin: the widespread perception of a lack of future ahead, at least within the home country. “There was no future there”, or “What future could I give them *[interviewee’s children]* there?”, are questions that recur in a number of narrative excerpts (cf. Carling, 2014). This resonates with what MacLeod (2009, p. 63), in an ethnography of youth groups in a disadvantaged housing estate, calls “a deeply entrenched cynicism about their future”. As one reads between the lines, cynicism is exactly the attitude of most interviewees towards their home country as they were about to leave it. It is also, in several cases, their prevailing way of framing homeland politics right now; a point which is often neglected in grand narratives of diasporas, migration and development.

However, as migrants’ life experience demonstrates, a deep-rooted sense of no-future-at-home need not entail a compression of individual aspirations. Rather, it may result in their *displacement*, if an *exit* option is available as a way ahead for cultivating them. “I wanted a different future for me and for my children” is, unsurprisingly, the kind of sentence which often matches with the ones above. How migrants’ perceived future is then shifted, re-signified and reshaped over time, and how (if at all) that “entrenched cynicism” is really overcome, are the central concerns of this article.

Leaving home is often reconstructed as a sudden, possibly painful decision – one that may have been accelerated by an equally sudden, unmissable opportunity to leave. “I just tried it, like so many others did”, is the exemplary presentation of Margherita, a 33 years old woman from Poland. Sometimes, as several instances suggest, the decision is communicated at the last moment to partners and even children. It is then narratively reconstructed as an abrupt breach of an otherwise unchanging life routine. While, with the benefit of hindsight, migration does entail a life course disruption, it is not necessarily perceived as such at the outset. An initial expectation of temporariness is systematically reported in these, and in so many more, emigrants’ stories (cf., among others, Cwerner, 2001; Morawska, 1987). That overseas permanence tends to be longer than anticipated is an issue that migrants sometimes struggle to come to terms with. How their aspirations are affected by the shift from a short- to a long-term life perspective abroad, hence by a fundamental change in “collectively expected duration” (Merton, 1984, p. 281), is also far from clear.

Whatever the case, the characteristics of Italy as a destination country are remarkably marginal among the reported motivations and aspirations of these interviewees. When asked what they knew of Italy before leaving, they tend to display limited and stereotypical knowledge. The question itself is perceived as little relevant. The choice of this country seems rather to reflect a perception of relatively weak immigration controls and decent job opportunities – at least in domestic work, which is marked by high demand and durable concentration of immigrant labour (Ambrosini, 2013). Rather than being a focus of aspirations in itself, the choice of the destination is typically a secondary or pragmatic concern, driven by interpersonal, generally kin-based social networks. Single critical events such as loss of job, debts or health problems in the family may also have a stake. The same holds for oppressive or violent family arrangements, particularly for women.

In a nutshell, the “migration aspirations” of immigrant domestic workers such as these interviewees are more *instrumental* than oriented to any specific place of destination (cf. Carling, 2014). What primarily nurtures them is the expected possibility to obtain better incomes and living conditions. The narrative of Lucio, a domestic worker from Peru, is a case in point on the future-oriented material targets at stake, against a background of relative deprivation, more than utter poverty:

My wife and I, we saw that the situation there was a bit… what we did aspire the most was to buy a house… with the money you got there you couldn’t do that much, you had a good living, I mean, but it was pretty difficult to buy a house. That’s why we have been – we have seen migration as a way of… working abroad, as a way of… getting something better. *(Lucio, Peruvian, 39, in Italy for 12 years)*

There is interestingly little, in the ways in which interviewees retell their earlier migrant experience, of aspirations as mere “dreams of fantasy” out of touch with reality. The latter are systematically attributed, instead, to non-migrants’ stereotypical understandings of migrants’ life conditions, which would grossly neglect their hardships. As Malù, a Filipina in her early forties, recalls:

I had no idea *[about Italy]*, they just told me that you gain well in Italy, and that there are a number of Filipinos… now, you know what’s the problem? I get angry with my co-nationals, coz every time I return home they *[Filipino non-migrants]* believe it’s a paradise here. You know, this is the problem. *(Malù, Filipina, 42, in Italy for 14 years)*

Accounts like this may be unnecessarily selective and self-benign in blaming other co-nationals for circulating all too rosy hopes about migration. There is no reason to exclude that incongruous expectations of the wages accessible abroad – to make the most obvious of examples – may have affected many individual decisions to leave. That said, these discordant attributions of naïf aspirations cast light on a more significant point: the social and community embeddedness of individual aspirations, including the aspiration to leave. While migration is typically an individual or household-based investment, driven by correspondingly “private” aspirations, it still takes place within a broader texture of pervasive social representations and shared aspirations (including the “depressed” ones, as in the previous instance of *no-future-here*). This can be easily detected in the comparative, possibly emulative bases of every single decision to leave. Now, the interaction between processes such as migrants’ remittances, the development of widespread aspirations to leave and the cumulative diffusion of a “culture of migration” is all too complex and case-dependent to be addressed here (see, among others, Ali, 2007; Belloni, 2015; Connell, 2008; Kandel & Massey, 2002). The fact remains that emigration backgrounds in home communities, including the previous infrastructural development of a migration system, are a significant influence on the individual and collective aspirations of prospective leavers.

### The future seen from the here-and-now: still a field for “upwards” aspirations?

Retracing migrants’ past aspirations from their present accounts, as in the previous section, is a slippery task – if one with few alternatives, here. There may be less of a risk, now, in exploring their present aspirations as future-related views, desires and fears. This mapping exercise, however, has also its challenges.

As interviewees are asked about the biographic changes associated with their migrant experience, *what* of their previous aspirations has been realized so far is often dubious or unclear. More clear and widespread is the emphasis on the personality and relational changes they have experienced over time. Once their present self-images and family attachments are compared with those of the past, immigrants’ self-representations share an emphasis on their personalities as more assertive, resilient and self-confident than “before”. A stronger capability to plan one’s own future is also stressed in several interviews – if only by contrast with the lack of long-term projects which some attribute to their left-behind counterparts. Their narratives abound with instances of social processes that could be framed, first, as *individualization* (e.g. Beck & Beck-Gernsheim, 2002), as suggested by the emphasis on greater economic and social autonomy, but also by the feelings of isolation from previous support networks; second, as *dissimilation* (Fitzgerald, 2014), a perception of increasing disalignment from non-migrant co-nationals in terms of lifestyles, mindsets and, possibly, in living conditions.^5^

As the focus is reset on respondents’ views of their own future, strong variations can be found in the spatial referents, temporal reach and emotional intensity of their aspirations. No single predominant configuration emerges out of them. What is clear, though, is that not all respondents would see “cultivating aspirations” as a worthwhile effort. Rather, their future-oriented aspirations reflect some mediation between present work circumstances that leave little room for improvement and long-term, often vague prospects of homecoming.

The very notion of home, while being emotionally nourished, may be hard to be emplaced in one specific location, as a lasting source of a better future (Boccagni, 2016b). In the words of Moroccan Farida, who takes some distance from her husband “who is a bit unhappy because he has always wished that there may be a future in our country”,

Future – This is something I’m thinking of, but there’s a long way ahead, it’s not easy. […] We don’t have a future yet, as if I can go to Morocco and stay for good – there is not a place that is like home for me, or for my children. You must start everything from scratch… I don’t think anybody wouldn’t like to go back, if you have a house to live in, a job to carry on… but I like Italy because it has given me something which my country has not, and it’s difficult to get it there. If there were a home and a job, I’d go back there. *(Farida, 28, in Italy for 11 years.)*

Although such uncertainties have much to do with poor prospects for improvement in home countries, they are also affected by the “ordinary” difficulties of low-skilled immigrant life at the *margins* of the receiving society, even when they are employed in *domestic* work. Over time, however, such living and working conditions can pave the way for substantial life improvements, at least in home communities. Particularly for pioneer or single migrant respondents, homeland turns out to be the main or even the only framework within which upwards aspirations are cultivated (for oneself, or at least for one’s children), against the little chances of social mobility abroad.

While the aspiration to return in the future is predominant, it is not shared by all respondents, though. The expected emplacement of the future does not depend only on the relative success of their immigrant trajectories, or on their living standards here and now. As, or possibly more significant is the reach and strength of intergenerational ties and obligations. This is most visible among female interviewees. In the meaningful formulation of Gheraldina, an Albanian woman with two children in their mid-twenties, “I’m not thinking of the future for me – I’m thinking [of it] for my children… future, by now, is for them”.

While first-generation adult respondents tend to frame homecoming as a desirable future option, this need not be the case for their children, whether reunited from the homeland or born abroad. For younger generations, returning home – hence, reframing homeland as a place for productive and biographic investments, rather than for enjoying old age – is generally an unlikely, undesirable development. “I can’t see so much future for her there”, says 48 years old Maria, thinking of her teenager daughter who was born and raised in Italy. If this is the case, however, migrants’ aspirations about their own future become irremediably more indeterminate, or at least “multi-sited”.

Two more aspects of this mixed configuration of aspirations are important here. First, in a number of cases aspirations tend to be framed in minimal and pragmatic terms, consistent with respondents’ segregated life and work circumstances. An aspirational curtailment, or downwards levelling, is at work here. Margherita’s reply to a question on her perceived future life prospects – “for now I’m content with what I have” – is an example, among many, of this creeping attitude. In a minority of interviews, however, a more radical stance emerges, which questions the commonsensical view of aspirations as something out there, just ready for use. Some narratives, particularly among women, point to the absence of conditions – i.e. external opportunities and internal dispositions – suitable to cultivate meaningful aspirations for the future. One of the worst psycho-social effects of immigration may then be a substantive deprivation of the interest, capability and orientation necessary to frame future as open to positive developments. “I muddle through day by day, without looking at the past, or even at the future – it’s always present”, is the self-presentation of Gloria, a Filipina of 38, against a question on “what has changed” over the two decades she has spent in Italy. The interview excerpts below are equally revealing of a fatalism that results, as time goes by, in decreasing capacities and interests to aspire.

I’ve no plan, no plan *[about the future]*. I’m one who lives day after day. You’d better live this way rather than doing plans all the time – what I’ll do the day after tomorrow, or in 10 years, or whatever. I’m just thinking that today was OK, tomorrow… we’ll see. *[…]* I’ve always said that I’ll be here as long as I can work – the day I can’t work anymore, I’ll go back. *(Mary, Ecuadorian, 45, in Italy for 10 years)*

I just don’t know *[what future will be like]*. We can’t talk about future, but we wish we’ll always be fine, don’t we? Let’s pray God for good health and quiet and serenity and then… other things, but most of all health. *(Vasilica, Rumanian, 53, in Italy for 11 years)*

[Q: *What do you imagine your future to be like? What do you think you will do?*]

Nothing.

*Q.: Are you looking for a job in another sector?*

No, no.

*Or always…?*

No, not even that.

*I mean, not even another domestic job.*

No. I’ve got a job now, I’m not looking for nothing more. When I haven’t it, I’ll look for another, if I want. *[…]*

*Have you ever looked for a job different from domestic or care work?*

No, never – don’t even know why! (*Mirela, Albanian, 41, in Italy for 15 years*)

This attitudinal mixture of vagueness, indecision and ostensible disinterest is a way of coping with the little room available for nourishing, let alone achieving, personal aspirations. It is also a reminder against the risk of conceiving people’s looks into the future as necessarily positive, optimistic or progressive. Particularly under precarious life circumstances, it should not come as a surprise that aspirations are levelled down or dismissed as irrelevant. The worried gaze into the future of the following middle-aged interviewees, well-aware of the dubious prospects for social security they will have when getting older (cf. Horn & Schweppe, 2015), is telling in this respect.

[Question: *What do you imagine your future like? What do you think you will do?*]

I imagine future to be horrible. I mean, even if I’ll succeed in getting a pension, I won’t have really much of it as a pension. What will I do then? I won’t have money enough to take a woman to care for me, either, if I get old. I don’t see a beautiful future. *(Bernadette, Madagascan, 45, in Italy for 20 years)*

To tell the truth, it *[the future]* makes me afraid. What will I do when old age comes? […] You know, I’m not 20 – I’m 47, and I’ve worked only for 15 in my country. I’ll get almost nothing there *[i.e. pension benefits]*, and here – when I can’t cope with it any more, I don’t know – I really don’t know. That’s what makes me feel afraid. *(Eva, Polish, 47, in Italy for 11 years)*.

At least in some cases, the pre-migration perception of “no future” in the country of origin is reproduced in the country of settlement, after a number of years spent there. Migration may then result in a way of curtailing aspirations, rather than nourishing them over time. At a minimum, the perceptions of one’s future can be open to (and possibly colonized by) all sorts of attitudes, moods and emotions. Aspirations as betterment-oriented constructs are by no means exhaustive of these stances.

There is a visible limit to the possibility of framing future as an open-ended “place” where all that is not available or achievable now can be projected. Such a limit has clearly to do with age and the life course, as well as with gendered expectations about family roles and obligations. It may be still more salient in live-in care work, which makes for a unique combination between spatial certainty – i.e. a highly segregated work setting – and temporal uncertainty – its duration being strictly connected with the survival of frail and care-dependent clients (Boccagni, 2016a).

## Immigrant domestic workers’ aspirations, unpacked

The next step of my analysis lies in retracing some communal patterns across the narratives of these interviewees, to be connected with the broader debate on aspirations. Is there anything like a typical “aspirational profile” out of their life circumstances, and how does it evolve over time? What insights can be drawn, once their subjective future as-seen-from-the-past is compared with future-as-seen-from-the-present? Last, what do these findings say for the study of the meanings and consequences of aspirations at large?

At first glance, and especially at the start of migration, interviewees’ aspirations are fundamentally *occupational* ones (Portes et al., 1978). Finding a job and higher wages is the key issue at stake. An in-depth reading of their narratives, though, shows a more complex and faceted aspirational profile. Employment in a richer country is less an aim in itself than the means towards individual and family wellbeing, thus involving other people (i.e. left-behind kin) and places (i.e. home communities). Adjectives such as *intergenerational* and *transnational* should also be used to capture the prevalent view-into-the-future of these immigrant domestic workers.

If occupational aspirations are “a mediating link between socioeconomic structures (what society offers) and individuals at the cultural level (what one wants)” (MacLeod, 2009, p. 22), respondents’ aspirations parallel this mediation and move it ahead, on a dual scale: over time, as they involve their descendants as (or even more than) themselves, and over space, as they try to connect host societies expected to be progressive, “modern” and open to self-fulfilment, and home societies regarded, by contrast, as unable or unwilling to provide opportunities or even only rights. Whether receiving societies meet such expectations, and whether the benefits accrued there can be imported back into home communities, is a case-dependent question which goes beyond the scope of this paper. The predominant tone of these respondents suggests pessimism in the regard, though.

While their initial experience of migration is typically underpinned by a view of the future as an empty, open and accessible space (cf. Adam & Groves, 2007), their present view of the future is both less open-ended and more uncertain. A friction emerges between relatively richer and emotionally intense aspirations *then*, while leaving home; and more modest, narrow and pragmatic, but sometimes still fuzzy aspirations *now*, after several years spent abroad. The proactive stance which arises in the accounts of their past futures – what Portes (2012) calls “the immigrant drive” – contrasts with the mixed and uncertain attitudes towards their present futures. The narrative of Emy, a Filipina with a relatively high length of stay in Italy, is a case in point.

Now, I feel a bit lonely sometimes… Earlier on, I had in my mind only one thought: to go ahead, improve my life – I had to be strong and work. I hadn’t this thought, “Oh my God, what’s happening to me?”. I was full of energy to work. Now instead, after so long, I’m starting to miss my country and be worried for my family, my daughter and husband. *(Emy, Filipina, 39, in Italy for 15 years)*

The contrast between *now* and *earlier on* cannot be reduced to an opposition between past wealth of aspirations and present lack of them. Uncertainty and ambivalence, more than pessimism, pervade respondents’ attitudes towards the future. Migration for low-skilled domestic work need not disempower personal aspirations. It nonetheless rearticulates and dovetails them, as immigrants’ life conditions are typically disadvantaged (by the standard of the host society) and hard to frame into hierarchical and linear categories such as “better” or “worse” (compared with the home society). While the immigrant condition itself is a source of lesser social status and relative deprivation, over time there seems to be also a cognitive adaptation to this. For most interviewees, individual aspirations decrease – at least for themselves, here and now. The same may not hold for their far future and for a range of significant others, most notably children, expected to benefit from immigrants’ striving (Boccagni, 2016a).

In a way, the individual aspirations of these immigrant domestic workers tend simply to become more realistic and better focused, based on the reality test of their everyday life. Migration seems to work as a multiplier of aspirations in its early stages, and even prior to its inception. Over time, instead, it acts as an aspirational regulator, or even a downwards leveller – at least for this particular immigrant profile, under the circumstances of the Italian labour market (Ambrosini, 2013).

With these premises, an analytical framework for dissecting migrant workers’ manifested aspirations – their everyday ways of “trying to get something better”, as an interviewee put it – could be developed along three key dimensions (cf. Spenner & Featherman, 1978):

- *Contents* (i.e. *aspiring what?*). This points to the impossibility of making sense of aspirations unless with regard to specific, subjectively meaningful and variable targets. This also calls for a focus on the sets of values, interests and rights in which aspirations are embedded, for instance regarding access to, or better conditions in, employment, income, education, family reunification etc.;
- *Relational references* (i.e., *to the benefit of whom?*). Aspirations need not be only ego-centred. One may cultivate aspirations involving primarily oneself as much as a variety of significant others, as in family-based migration flows such as those at stake here;
- *Space-time horizons* (i.e. *where,* and *when?*). There is nothing obvious either in the *spatial* frames underlying aspirations (i.e. migrants’ home and/or host societies), or in the *temporal* ones, that is, the social durations involved – whether an expectedly short-term one, a long-term one (e.g. retirement age or the adulthood of the next generations), or possibly a temporally undetermined one, whenever migrants’ perceived future horizons are little defined.

At all of these levels, migration-related aspirations call for a new and more sophisticated understanding. That aspirations are time-dependent (hence call for diachronic and life-course related research), and that they are affected by a variable combination of micro-, meso- and macro- factors, is important. It is not the whole story, though. As important is to dissect, in individuals’ ways of cultivating aspirations, the coexistence and possibly the conflict between different aspired values, driven by distinct kinship regimes and moral economies, as much as by personal preferences. Likewise, one should appreciate the interdependence between personal aspirations and the life prospects of a number of significant others, so that any exclusively individual(istic) view is bound to be oversimplistic. Following these insights, my framework points to the need to go beyond a linear and cardinal understanding of aspirations as *high* vs *low*, or as a principled reference to something *better* or *more* than what used to be there. Different subjective criteria and dimensions of analysis need to be brought into the equation – with all of the attendant tensions and negotiations between the relevant actors. At the same time, the lowering aspiration trend that emerges across these interviews is inseparable from respondents’ socio-demographics. There is little surprise in a prevalence of modest and pragmatic aspirations among middle-aged women involved in intensive care work and in equally “sticky” moral and economic obligations towards their family members left behind (Lutz, 2011; Parrenas, 2015). The weight of their work socialization in Italy should not go unnoticed either. That immigrants’ everyday life is *not* an amplifier of aspirations, here, is also a social and ethnic capital effect: one resulting from strong occupational segregation, as well as from the prevalence of informal mechanisms of bounded solidarity along narrowly ethnic lines (Catanzaro & Colombo, 2009).

Having said this, one might wonder whether *aspirations matter*, as a research topic, even when they are not accomplished. Based on my case study, the response cannot but be positive. Researching aspirations and following up their tentative achievement is a worthwhile effort because they still mirror migrants’ desired future, *and* their relative autonomy and efficacy in shaping it. Migrants’ aspirations are unique as a research window into the social factors which affect their agency and its transformation over time. Both pursuit and transformation are related to one’s evolving and changing terms of reference – that is, the perceived life standards and opportunities available in host, and/or in home societies.

## Conclusion

Aspirations, as subjective and volitional views of the future, are worth understanding in light of their evolution over time – as long as retrospective life histories enable this, if only for the lack of viable alternatives on a large scale. Comparing “then” and “now”, within the narratives of these first-generation immigrant domestic workers, points to a redistribution of aspirational trajectories into a variety of arrangements. Rather than being invariably the same, respondents’ aspirations are displaced, deferred, intergenerationally invested and possibly curtailed over time – while also remaining, more often than not, remarkably blurred. While the aspiration to leave in the first place is quite clear-cut, widespread and socially (as well as morally) legitimated, it gradually leaves space to more fuzzy, contingent and reversible micro-aspirations about immigrants’ personal and family future.

To the typically middle-aged, mostly female domestic workers considered here, the migration experience turns out to be an aspirational leveller, or a source of downwards aspirations, as far as the occupational domain is concerned. This is consistent with the limited opportunities available for better (or even only different) jobs, with their prevalent lack of valuable human and social capital, and with their functional adaptation to the domestic work niche. Seldom, if ever, do their narratives show any success in searching for better work conditions or different sectors of employment. This is little of a surprise for Italy, in a sector – such as domestic work – even less unionized and permeable to upwards mobility than the others in which immigrant labour is concentrated (Marchetti, 2012).

Yet, an understanding of migrant aspirations along these lines only, and only from the viewpoint of the receiving society, would be narrow and simplistic. A broader, longer-term and multi-sited biographic optic should be pursued instead. Along these lines, the migrant experience has still a potential to nourish aspirations of self-achievement and socio-economic mobility for oneself, or at least for children and closer kin. Such a potential is shaped by generational and life course circumstances and is not at odds with their poor *occupational* aspirations within domestic work. The contradiction between family-oriented and occupational aspirations should however be revisited in the case of second generation immigrants, which is still to be investigated, in Italy, in this optic. It is there that the aspirational gap between generations, and between desires and accomplishments, is more likely a source of tensions, and deserves further research.

Altogether, subjective aspirations cannot be underestimated as a biographically relevant set of attitudes and representations “ahead”; hence, a meaningful window into migrants’ perceived future and their stances towards it. While much of the debate on immigrant integration focuses on the (expected) outcomes, a better understanding of the underlying everyday *processes* – and of the ways immigrants understand and try to shape them in aspirational terms – is in order to refine the understanding of integration itself.

## Footnotes

1 Exceptions include Appadurai (2004), as mentioned above.

2 The key topics inside the interview guide are as follows: Family and social background; Work and everyday life in the country of origin; Development of the migration project; Migration pathways; Current job and work history in Italy; Everyday life in Italy; Welfare needs and interactions; Transnational relationships; Future-related changes and perspectives; Key socio-demographics. All in-depth interviews, generally lasting from 2 to 4 h, were conducted in Italian by specially trained interviewers.

3 While the original research design was driven by a “purposeful sampling” logic, it still followed a number of selection criteria, resulting in pre-established quotas along lines of territorial distribution, gender, length of stay and condition of employment (Catanzaro & Colombo, 2009).

4 Quoting from Luhmann (1976).

5 Importantly, the feeling of being increasingly different to co-nationals at home need not go hand-in-hand with a stronger feeling of assimilation into the receiving society.
